# The Good Life: Assessing the Relative Importance of Physical, Psychological, and Self-Efficacy Statuses on Quality of Well-Being in Osteoarthritis Patients

**DOI:** 10.1155/2013/914216

**Published:** 2013-12-25

**Authors:** Charles Van Liew, Maya S. Santoro, Arielle K. Chalfant, Soujanya Gade, Danielle L. Casteel, Mitsuo Tomita, Terry A. Cronan

**Affiliations:** ^1^San Diego State University, 6505 Alvarado Road, Suite 110, San Diego, CA 92120, USA; ^2^San Diego State University and University of California, Joint Doctoral Program in Clinical Psychology, San Diego, CA 92120, USA; ^3^Kaiser Permanente of Southern California, Panorama City, Los Angeles, CA 91402, USA

## Abstract

*Background and Purpose*. The purpose of the present study was to examine the interrelationships among physical dysfunction, self-efficacy, psychological distress, exercise, and quality of well-being for people with osteoarthritis. It was predicted that exercise would mediate the relationships between physical dysfunction, self-efficacy, psychological distress, and quality of well-being. *Methods*. Participants were 363 individuals with osteoarthritis who were 60 years of age or older. Data were collected from the baseline assessment period prior to participating in a social support and education intervention. A series of structural equation models was used to test the predicted relationships among the variables. *Results*. Exercise did not predict quality of well-being and was not related to self-efficacy or psychological distress; it was significantly related to physical dysfunction. When exercise was removed from the model, quality of life was significantly related to self-efficacy, physical dysfunction, and psychological distress. *Conclusions*. Engagement in exercise was directly related to physical functioning, but none of the other latent variables. Alternatively, treatment focused on self-efficacy and psychological distress might be the most effective way to improve quality of well-being.

## 1. The Interrelationships of Self-Efficacy, Psychological Distress, Physical Dysfunction, Exercise, and Quality of Well-Being among People with Osteoarthritis

Osteoarthritis (OA) is a joint disorder, characterized by degeneration of cartilage creating joint pain and stiffness that worsen over time, most often affecting the hips and knees and leading to disability [1–3]. OA is the most common form of arthritis and affects close to 27 million Americans [4, 5]. After the age of 65, 60% of men and 70% of women experience OA [6]. OA is a leading cause of chronic pain, disability, and functional impairments [6]. Besides joint replacement, the most effective treatments available for OA consist of a combination of pharmacotherapy and behavioral self-management techniques [7]. Behavioral interventions have been shown to reduce the severity of symptoms associated with OA [8–10]. Behavioral treatments are largely focused on pain reduction and management and facilitation of mobility and physical functioning [11]. However, several factors affect the success of these treatments, including exercise, physical dysfunction, self-efficacy, and psychological distress [11]. These factors have been examined individually for their impact on quality of well-being in the OA population but have not been examined simultaneously.

Physical exercise has become widely recommended for individuals with OA [12], because it has been related to longevity [13]. Devos-Comby et al. [11] conducted a meta-analysis on treatments for OA and found that exercise programs reduced pain, improved physical functioning, and enhanced quality of life among individuals with OA. Despite this, close to 44% of adults with arthritis report not engaging in exercise [6].

When mobility and physical functioning are impaired, individuals are less likely to engage daily activity. People diagnosed with arthritis report less daily physical activity than those without arthritis [6]. The Center for Disease Control (CDC) reported that approximately 80% of adults with OA have some movement limitations that affect daily activities [1]. Physical dysfunction is related to reduced quality of life and lower self-efficacy [14–17], which is defined as a person's belief in his/her ability to influence events that affect his/her life [18, 19]. Increased self-efficacy for physical activity is associated with increased participation in exercise for people with arthritis [20, 21]. Having high levels of self-efficacy is associated with higher quality of life, decreased pain, and increased activity among people including those with OA [22–24].

Psychological distress is another factor that is associated with exercise and quality of life among people with OA [25, 26]. Evidence suggests that anxiety and depression are related to reduced functioning and to lower levels of physical activity among the OA populations [26, 27]. Although depression may pose barriers to activity engagement, physical activity has been shown to improve its symptoms [27] and is a common focus of behavioral therapies (e.g., behavioral activation). Alternatively, improvements in depression are also likely to lead to increases in activity levels and quality of life [28].

The purpose of the present study was to examine the interrelationships among physical dysfunction, self-efficacy, psychological distress, exercise, and quality of life among people with older adults with OA using structural equation modeling. These variables have not been assessed concurrently in an older OA population. It was hypothesized that physical dysfunction, psychological distress, and self-efficacy all would predict probability of participating in exercise uniquely and that participation in exercise would mediate the effect of each of these on quality of well-being.

## 2. Method

### 2.1. Participants

Participants were 363 members (*N* = 233 women, *N* = 130 men) of a large health maintenance organization (HMO) in Southern California who were 60 years of age or older (*M*_age_ = 69, SD = 5.6) and had a physician's diagnosis of osteoarthritis (OA) that was confirmed with radiographic evidence within the individual's medical file. The participants were primarily Caucasian (92.3%), married (72.7%), and retired (75%). Nearly 29% of participants reported having completed a high school education or equivalent, 40.2% reported several years of college education, and 25.4% had obtained higher degrees or other professional certificates. Participants' median annual income ranged from $20,000 to $30,000. See Table 1 for additional demographic information.

### 2.2. Measures

#### 2.2.1. Demographic Variables

Participants were asked to provide a brief demographic history, which included their age, gender, education level, employment, income, marital status, and date of diagnosis.

#### 2.2.2. Arthritis Impact Measurement Scale (AIMS)

The AIMS is a disease-specific measure of health status for people with arthritis. The scale is self-administered and consists of 57 questions categorized into nine subscales: mobility, physical activity, dexterity, social role, social activity, activities of daily living, pain, depression, and anxiety. Internal reliability for each of the subscales ranges from *α* = .63 to .88 [29].

#### 2.2.3. Quality of Well-Being (QWB) Scale

The QWB scale was used to assess global quality of well-being. The QWB scale evaluates the participant's functioning and symptoms for the 6 days prior to the assessment [30]. Its three subscales are mobility, physical activity, and social activity. The QWB scale has been shown to be a valid and reliable instrument for assessing health outcomes in a general elderly population and in a population with specific chronic or disabling conditions [30].

#### 2.2.4. Center for Epidemiologic Studies Depression Scale (CES-D)

The CES-D was designed to measure current levels of depressive symptoms, with an emphasis on depressed mood [31]. The CES-D is a 20-item self-report measure designed to assess depression in nonpsychiatric populations. Studies indicate that the scale is internally consistent, has moderate test-retest reliability, and has high concurrent and construct validity (e.g., 30).

#### 2.2.5. The Arthritis Self-Efficacy Scale (ASES)

The ASES consists of 20 items that require respondents to indicate how certain they are that they can perform various tasks on a scale from 10 (very uncertain) to 100 (very certain), with higher scores indicating higher self-efficacy [16]. Sample items include “how certain are you that you can manage arthritis pain during your daily activities?” and “how certain are you that you can turn an outdoor faucet all the way on and all the way off?” The questionnaire consisted of three subscales: pain, function, and other symptoms. Lorig et al. [16] found that subscale reliability was .87 for pain, .85 for function, and .90 for other symptoms.

#### 2.2.6. Arthritis Helplessness Index (AHI)

The AHI was developed by Stein et al. [32]. The questionnaire consists of 15 items, scaled in a 6-point Likert format from strongly disagree (1) to strongly agree (6). Participants were asked whether they agreed or disagreed with statements like, “I have considerable ability to control my pain” and “it seems as though other factors beyond my control affect my arthritis.” Cronbach's alpha indicated overall internal reliability of .69 and test-retest reliability of .52 over a 1-year period. Internal consistencies for the two subscales, as assessed by Cronbach's alpha, were .75 for the internality factor and .63 for the helplessness factor [32].

#### 2.2.7. Exercise

Participants were asked to indicate whether or not they participated in exercise.

### 2.3. Procedure

The data for this study were collected during the baseline assessment period prior to participants engaging in a social support and education intervention. To be eligible to participate in the present study, participants had to be 60 years of age or older, have a diagnosis of OA, and be willing and able to attend 10 weekly and 10 monthly meetings over a course of 1 year. Three thousand potential participants were randomly selected from the total population of 50,450 HMO members in San Diego County. Because the prevalence of OA in this population is approximately 50% of those over the age of 60, we expected 1,500 of those contacted to be eligible to participate. Three hundred and sixty-three of the 3,000 HMA members that were contacted by mail volunteered to participate in a larger study and completed the battery of questionnaires.

### 2.4. Analytic Procedure

Statistical analyses were performed using Stata 12.1. A series of structural equation models (SEMs) using full information maximum likelihood (FIML) was used to test the relationships among self-efficacy, psychological distress, physical dysfunction, exercise, and quality of well-being. The primary observed response variable was quality of well-being (QWB). The latent explanatory variables were (1) self-efficacy (SE), (2) psychological distress (PSYCH), and (3) physical dysfunction (PHYS). The binary mediator was self-reported exercise (EX). No changes were made to the measurement factor loading or structural pathways within models; however, as determined by modification indices and conceptual reasoning, error covariances were added to improve model fit. This strategy was decided *a priori* based upon the likely high interrelatedness of many of these constructs and their components.

In order to examine the effects of the explanatory variables on QWB in this sample of individuals with OA, the model fit (using descriptive indices of model fit (e.g., Comparative Fit Index and root mean squared error of approximation)), the standardized factor loadings, and the specific tests for the factor loadings were assessed. Overall model fit was determined using the recommendations of Bentler [33]. Although the likelihood ratio *χ* ^2^ is reported, this inferential test performs poorly as a sole determinant of model fit [33]. Therefore, in the current study, the Comparative Fit Index (CFI; 33) and the root mean square error of approximation (RMSEA; 34) were interpreted as measures of descriptive fit. Both the CFI and RMSEA are standardized measures of descriptive model fit that range in value from 0 to 1. For the CFI, values greater than .95 indicate a reasonable model, and values greater than .90 indicate a plausible model. For the RMSEA, values less than .08 indicate acceptable model fit, and values less than .05 indicate good model fit.

## 3. Results

### 3.1. Measurement Models for Latent Variables

The measurement models for PHYS, PSYCH, and SE fit well statistically, *χ* ^2^ (55, *N* = 363) = 51.12, *P* = .6237; *χ* ^2^ (2, *N* = 363) = .74, *P* = .6896; *χ* ^2^ (2, *N* = 363) = 2.87, *P* = .2377, and descriptively, CFI = 1.00, RMSEA < .0001; CFI = 1.00, RMSEA < .0001; CFI = .998, RMSEA = .035, respectively. See Tables 2 and 3 for loadings and covariances, respectively, for the measurement models. The vast majority of the error covariances were subsumed in the PHYS measurement model, because individual items (not scales or subscales) were used to construct this latent variable.

### 3.2. Full, Mediated Model

The full model was constructed to model the effects of PHYS, PSYCH, and SE on QWB via the mediator, EX. The model did not fit statistically, *χ* ^2^ (248, *N* = 363) = 888.04, *P* < .0001, or descriptively, CFI = .790, RMSEA = .084, AIC = 32109.794, and BIC = 32507.023. In order to permit interpretation of model coefficients, modification indices (MIs) were obtained to improve model fit via alterations in error covariances. Covariances with MIs of greatest value were added singularly, provided that the covariances were conceptually tenable. For the sequential list of added covariances, see Table 3. After five covariances were added, the descriptive fit of the model was adequate, CFI = .929, RMSEA = .050, AIC = 31691.573, and BIC = 32108.274; although, the statistical fit was lacking still, *χ* ^2^ (243, *N* = 363) = 459.82, *P* < .0001. Based upon the adequate descriptive fit, interpretation of the model coefficients followed.

The measurement models remained sound within the structural model (see Table 4). The majority of covariances remained statistically significant (see Table 5). Examining the structural pathways, the relationship between EX and QWB was not statistically significant, *B* = .0718, *P* = .171. Neither were the relationships between PSY or SE and EX, *B* = −.0138, *P* = .081; *B* = .0653, *P* = .217, respectively. In fact, the bivariate correlation between the observed variables, EX and QWB, was nonsignificant, *r* = .0713, *P* = .1756. The only significant structural coefficient was the relationship between PHYS and EX, *B* = −.1748, *P* = .005. Thus, as physical dysfunction scores increased (demonstrating increased physical complications), the probability of participating in exercise decreased. On the whole, this model demonstrates that, in our sample of OA participants, only physical dysfunction (and not self-efficacy or psychological distress) was related to exercise, and exercise was not related to quality of well-being.

### 3.3. Nonmediated Structural Model

Based on the previous model, EX was eliminated from the model to determine whether PHYS, PSY, and SE uniquely and significantly contributed to QWB. In this model, the MI changes entered into the previous model were maintained, with the exception of the covariances that related to QWB, because QWB was exogenous in the nonmediated model (see Table 8 for all error covariances). This model did not fit statistically, *χ* ^2^ (222, *N* = 363) = 406.34, *P* < .0001, but it did fit well descriptively, CFI = .939, RMSEA = .048, AIC = 31132.477, BIC = 31521.918, and CD = .827. The measurement models remained intact (see Table 6), and the covariances remained consonant with previous models (see Table 7). The structural model (QWB→SE, PHYS, PSYCH) demonstrated that physical dysfunction, psychological distress, and self-efficacy were related largely and significantly to QWB, *B* = −.7910, *P* < .0001; *B* = −.2852, *P* < .0001; *B* = .4267, *P* < .0001, respectively. These relationships are in the expected directions, with greater physical impairment relating to lower QWB, greater psychological impairment relating to lower QWB, and greater self-efficacy relating to higher QWB. Both Akaike's and the Bayesian Information Criteria support the superiority of this model to the model that includes EX as a mediator.

## 4. Discussion

In this study, structural equation modeling was used to determine whether exercise mediated the relationships among self-efficacy, physical dysfunction, psychological distress, and QWB and to examine the interrelationships among these variables. The results indicated that self-efficacy and psychological distress did not relate to engagement in exercise; only level of physical dysfunction was related to engagement in exercise. In addition, exercise was not related to one's QWB. However, physical dysfunction, psychological distress, and self-efficacy each were independently related to health status. These findings are consistent with past research and illustrate the importance of these factors in health status [16, 34].

Exercise was related to physical dysfunction, but because of the study's cross-sectional design, we do not know whether physical dysfunction impaired one's ability to exercise, whether lack of exercise increased physical dysfunction, or whether the relationship was bidirectional. Longitudinal studies are needed to determine the direction of the relationships to better inform treatment efforts. Physical dysfunction was related to self-efficacy over arthritis, which was also related to psychological distress. That is, worse physical dysfunction was related to lower self-efficacy, and heightened psychological distress was also related to lower self-efficacy. Thus, it appears that exercise is not as important a predictor of quality of life among older people with OA as other factors. One explanation for this finding is that older people with OA may believe that their physical health is unchangeable or is worsened by exercise. Another explanation may be that they believe that their quality of life is only well managed by other mechanisms, such as medication.

People who experience greater physical impairment because of their chronic condition are less likely to engage in activities that might improve their condition and more likely to experience psychological distress [28]. The present study suggests that we need to identify the pathways that self-efficacy, psychological distress, and physical functioning take to affect changes in QWB among older people with OA. The results from this study indicate that the pathway to affect QWB may not include exercise. Researchers may be well advised to develop interventions directly focused on improving self-efficacy and physical functioning and decreasing psychological distress to improve QWB.

In the present study, exercise was not related to QWB. The measure of QWB used in this study assessed mobility, physical activity, and social activity. Because physical functioning was related to mobility and physical functioning, it is not surprising that physical functioning was directly related to QWB. However, the fact that exercise was not related to the QWB calls into question the goals of treating OA. Is the goal of treating OA to improve quality of life or to increase longevity? If longevity is the goal, then treatment programs should focus on increasing exercise. On the other hand, if quality of well-being is the priority, then treatment might be most effective when it is focused directly on affecting self-efficacy, physical dysfunction, and psychological distress. The participants in this study had a mean age of over 69. It could be that increasing quality of life is more important for older people with OA, or for others living with pain-related conditions, than is increasing longevity. The model suggests that QWB in older adults with OA is predicted by a person's physical functioning, psychological status, and self-efficacy, but not their engagement in exercise.

The present study also showed that physical dysfunction did not affect quality of life through exercise. Thus, the challenge may be how does one increase mobility and independence while decreasing pain and stiffness, if not through exercise? Perhaps activities that are not classified as “exercise” are part of the answer. It is possible that being active and getting out, but not necessarily “exercising,” are key to physical health as they relate to quality of life in this population of older individuals with OA.

One limitation of this study is that “exercise” was assessed by a single yes/no question that asked whether or not the participant exercised. No definition of exercise was given to the participant; therefore, participants may have defined “exercise” in various ways, which may partially account for the findings. It should be noted that the exercise variable was significantly correlated with participants' metabolic equivalent of task (MET) expenditure at later time points within the intervention. However, future studies should include a more comprehensive evaluation of exercise and seek to determine whether this type of model is invariant across various OA patient subgroups.

In summary, the relationships among self-efficacy, psychological distress, physical dysfunction, exercise, and quality of well-being are important factors to consider in treating people with OA. As the mean age of our population increases and OA becomes more prevalent in the population, more research is needed to determine how to effectively design interventions/treatments to *improve* life for those with OA.

## Figures and Tables

**Table 1:** Participant demographic and clinical characteristics.

Item	Valid %	*N*
Gender		
Male	35.81	130
Female	64.19	233

Ethnicity		
White	92.29	335
Hispanic	2.75	10
Black	1.65	6
Other	1.65	6
Decline to state	1.10	4

Age		
59 to 69 years	56.47	205
70 to 79 years	40.77	148
>79 years	2.75	10

Relational status		
Single	4.96	18
Married	72.73	264
Widowed	14.33	52
Divorced	7.99	29

Education		
High school graduate or less	31.13	113
Some college/trade school	22.31	81
Bachelor's degree	19.28	70
Graduate level degree	23.97	87
Decline to state	3.31	12

Family income		
$19,999 or less	24.24	88
$20,000–$39,999	38.29	139
$40,000–$59,999	17.36	63
$60,000 or more	8.82	32
Decline to state	11.29	41

Employment status		
Part-time	17.08	62
Full-time	75.21	273
Retired/unemployed	7.72	28

Length of diagnosis		
Less than 5 years	30.85	112
5–10 years	27.82	101
10–15 years	19.56	71
15–20 years	6.89	25
More than 20 years	2.20	8
Not reported	12.67	46

**Table 2:** Standardized factor loadings for measurement models.

Latent	Observed	*B*	SE	|*z*|	*P*	95% CI LB	95% CI UB
PHYS	WEIGHT	.1988	0611	3.25	.001	.0790	.3186
PHYS	TROUBE	−.5993	.0538	11.15	<.001	−.7047	−.4940
PHYS	ASSWA	−.1906	.0641	2.97	.003	−.3163	−.0650
PHYS	TROUWO	−.5473	.0576	9.50	<.001	−.6603	−.4343
PHYS	JOINTP	−.4085	.0566	7.22	<.001	−.5195	−.2976
PHYS	AMOVE	.1743	.0617	2.83	.005	.0534	.2952
PHYS	TROUWM	−.6984	.0504	13.87	<.001	−.7971	−.5997
PHYS	LIMITA	−.5866	.0537	10.91	<.001	−.6919	−.4812
PHYS	SEREP	−.4089	.0568	7.20	<.001	−.5202	−.2976
PHYS	PAIN	−.4262	.0556	7.67	<.001	−.5351	−.3173
PHYS	STIFF	−.2989	.0616	4.85	<.001	−.4196	−.1782
PHYS	ASSIST	−.2422	.0612	3.96	<.001	−.3621	−.1222
PHYS	STAYIN	−.2449	.0610	4.02	<.001	−.3644	−.1254
PHYS	INBED	−.2179	.0674	3.23	.001	−.3499	−.0858
PSYCH	CESD	.8000	.0229	34.97	<.001	.7552	.8449
PSYCH	AIMD	.9565	.0161	59.54	<.001	.9250	.9880
PSYCH	AIMA	.7983	.0231	34.59	<.001	.7531	.8435
PSYCH	AIMIS	.3340	.0488	6.84	<.001	.2383	.4296
SE	EFFPAIN	.3340	.0684	4.89	<.001	.2000	.4680
SE	EFFACT	.4804	.0675	7.12	<.001	.3481	.6127
SE	EFFSYM	.8852	.0579	15.29	<.001	.7718	.9987
SE	ARTHINT	−.5022	.0512	9.81	<.001	−.6026	−.4012
SE	ARTHHEL	−.6061	.0520	11.65	<.001	−.7080	−.5042

Note: SE: standard error; 95% CI LB: 95% confidence interval lower bound; 95% CI UB: 95% confidence interval upper bound.

**Table 3:** Standardized error covariances within measurement model.

Latent	First OV	Second OV	*r*	SE	|*z*|	*P*	95% CI LB	95% CI UB
PHYS	WEIGHT	AMOVE	−.1162	.0528	2.20	.028	−.2196	−.0128
PHYS	WEIGHT	ASSIST	.1422	.0428	3.32	.001	.0583	.2261
PHYS	TROUBE	LIMITA	.2229	.0702	3.17	.001	.0853	.3605
PHYS	ASSWA	TROUWM	.1171	.0483	2.42	.015	.0224	.2119
PHYS	ASSWA	ASSIST	.5049	.0381	13.26	<.001	.4303	.5795
PHYS	ASSWA	STAYIN	.3743	.0448	8.36	<.001	.2865	.4621
PHYS	ASSWA	INBED	.3577	.0456	7.85	<.001	.2684	.4470
PHYS	TROUWO	TROUWM	.3524	.0675	5.22	<.001	.2200	.4847
PHYS	TROUWO	STAYIN	.1808	.0434	4.17	<.001	.0958	.2658
PHYS	TROUWO	INBED	.1560	.0541	2.89	.004	.0500	.2620
PHYS	JOINTP	SEREP	.4069	.0474	8.59	<.001	.3140	.4997
PHYS	JOINTP	PAIN	.4324	.0426	9.35	<.001	.3418	.5231
PHYS	JOINTP	STIFF	.2200	.0527	4.18	<.001	.1168	.3232
PHYS	AMOVE	ASSIST	−.2176	.0415	5.25	<.001	−.2989	−.1364
PHYS	TROUWM	INBED	.1362	.0618	2.20	.028	.0151	.2574
PHYS	LIMITA	STIFF	.1069	.0517	2.07	.039	.0055	.2083
PHYS	SEREP	PAIN	.6062	.0356	17.01	<.001	.5363	.6760
PHYS	SEREP	STIFF	.3076	.0501	6.13	<.001	.2093	.4059
PHYS	PAIN	STIFF	.3140	.0502	6.26	<.001	.2157	.4123
PHYS	ASSIST	STAYIN	.4435	.0411	10.80	<.001	.3631	.5240
PHYS	ASSIST	INBED	.3065	.0460	6.66	<.001	.2163	.3966
PHYS	STAYIN	INBED	.3476	.0458	7.59	<.001	.2578	.4374
SE	EFFPAIN	EFFSYM	.2637	.1009	2.61	.009	.0659	.4615
SE	EFFPAIN	ARTHINT	−.2836	.0534	5.31	<.001	−.3884	−.1789
SE	EFFACT	EFFSYM	.3517	.1061	3.31	.001	.1436	.5597

Note: OV: observed variable; SE: standard error; 95% CI LB: 95% confidence interval lower bound; 95% CI UB: 95% confidence interval upper bound.

**Table 4:** Modification-indicated covariance additions.

First	Second	MI	*P*	Std. EPC	Δ*χ* ^2^	ΔCFI	ΔRMSEA
PHYS	SE	81.251	<.001	−.6239	−123.05	.040	−.008
SE	EFFACT	58.382	<.001	−.7277	−63.04	.020	−.003
QWB	PHYS	79.748	<.001	−.4369	−120.41	.040	−.011
QWB	SE	65.284	<.001	.3696	−72.18	.023	−.007
PSY	SE	33.791	<.001	−.2146	−49.54	.016	−.005

Note: MI: modification index; Std. EPC: standardized expected parameter change; CFI: comparative fit index; RMSEA: root mean squared error of approximation.

**Table 5:** Measurement models within full, mediated structural model.

Latent	Observed	*B*	SE	|*z*|	*P*	95% CI LB	95% CI UB
PHYS	WEIGHT	.1287	.0557	2.31	.021	.0196	.2378
PHYS	TROUBE	−.5735	.0411	13.97	<.001	−.6540	−.4930
PHYS	ASSWA	−.2657	.0523	5.08	<.001	−.3682	−.1632
PHYS	TROUWO	−.5273	.0429	12.30	<.001	−.6114	−.4433
PHYS	JOINTP	−.4465	.0465	9.61	<.001	−.5375	−.3554
PHYS	AMOVE	.1698	.0544	3.12	.002	.0631	.2765
PHYS	TROUWM	−.6227	.0394	15.82	<.001	−.6999	−.5455
PHYS	LIMITA	−.5401	.0427	12.65	<.001	−.6238	−.4565
PHYS	SEREP	−.4538	.0460	9.87	<.001	−.5439	−.3637
PHYS	PAIN	−.4872	.0443	10.99	<.001	−.5740	−.4003
PHYS	STIFF	−.3233	.0507	6.37	<.001	−.4228	−.2239
PHYS	ASSIST	−.3295	.0502	6.56	<.001	−.4279	−.2311
PHYS	STAYIN	−.3486	.0497	7.02	<.001	−.4459	−.2512
PHYS	INBED	−.3311	.0503	6.58	<.001	−.4297	−.2326
PSY	CESD	.8137	.0215	37.84	<.001	.7716	.8559
PSY	AIMD	.9353	.0154	60.85	<.001	.9052	.9654
PSY	AIMA	.8113	.0217	37.41	<.001	.7688	.8538
PSY	AIMIS	.3448	.0488	7.06	<.001	.2491	.4405
SE	EFFPAIN	.3032	.0577	5.26	<.001	.1902	.4163
SE	EFFSYM	.8462	.0373	22.70	<.001	.7731	.9193
SE	EFFACT	.8760	.0668	13.12	<.001	.7451	1.0068
SE	ARTHINT	−.4574	.0458	9.98	<.001	−.5473	−.3675
SE	ARTHHEL	−.6315	.0410	15.40	<.001	−.7118	−.5511

Note: OV: observed variable; SE: standard error; 95% CI LB: 95% confidence interval lower bound; 95% CI UB: 95% confidence interval upper bound.

**Table 6:** Standardized error covariances within full, mediated structural model.

Latent	First	Second	*r*	SE	|*z*|	*P*	95% CI LB	95% CI UB
PHYS	WEIGHT	AMOVE	−.1017	.0522	1.95	.051	−.2040	.0005
PHYS	WEIGHT	ASSIST	.1369	.0430	3.18	.001	.0525	.2212
PHYS	TROUBE	LIMITA	.2701	.0530	5.10	<.001	.1663	.3739
PHYS	ASSWA	TROUWM	.0987	.0416	2.37	.018	.0172	.1801
PHYS	ASSWA	ASSIST	.4871	.0388	12.55	<.001	.4111	.5632
PHYS	ASSWA	STAYIN	.3545	.0455	7.79	<.001	.2653	.4437
PHYS	ASSWA	INBED	.3354	.0455	7.79	<.001	.2438	.4270
PHYS	TROUWO	TROUWM	.4071	.0468	8.69	<.001	.3154	.4989
PHYS	TROUWO	STAYIN	.1623	.0422	3.85	<.001	.0796	.2449
PHYS	TROUWO	INBED	.1130	.0497	2.27	.023	.0156	.2103
PHYS	JOINTP	SEREP	.3802	.0465	8.17	<.001	.2890	.4713
PHYS	JOINTP	PAIN	.4009	.0458	8.75	<.001	.3112	.4907
PHYS	JOINTP	STIFF	.1996	.0512	3.89	<.001	.0991	.3000
PHYS	AMOVE	ASSIST	−.2219	.0417	5.32	<.001	−.3036	−.1401
PHYS	TROUWM	INBED	.0797	.0507	1.57	.116	−.0196	.1791
PHYS	LIMITA	STIFF	.1054	.0481	2.19	.028	.0111	.1998
PHYS	SEREP	PAIN	.5829	.0358	16.30	<.001	.5128	.6530
PHYS	SEREP	STIFF	.2885	.0488	5.91	<.001	.1929	.3842
PHYS	PAIN	STIFF	.2926	.0489	5.99	<.001	.1968	.3883
PHYS	ASSIST	STAYIN	.4167	.0420	9.93	<.001	.3345	.4990
PHYS	ASSIST	INBED	.2750	.0472	5.83	<.001	.1826	.3674
PHYS	STAYIN	INBED	.3118	.0475	6.56	<.001	.2187	.4050
SE	EFFPAIN	EFFSYM	.2621	.0714	3.67	<.001	.1221	.4021
SE	EFFPAIN	ARTHINT	−.2832	.0486	5.83	<.001	−.3784	−.1880
SE	EFFACT	EFFSYM	.3225	.0662	4.87	<.001	.1928	.4523
	PHYS	SE	−.6868	.0486	14.13	<.001	−.7820	−.5915
	SE	EFFACT	−.4443	.0604	7.36	<.001	−.5626	−.3260
	QWB	PHYS	−.7920	.0361	21.96	<.001	−.8627	−.7213
	QWB	SE	.3476	.0462	7.52	<.001	.2570	.4381
	PSY	SE	−.3408	.0524	6.51	<.001	−.4435	−.2381

Note: OV: observed variable; SE: standard error; 95% CI LB: 95% confidence interval lower bound; 95% CI UB: 95% confidence interval upper bound.

**Table 7:** Measurement models within nonmediated structural model.

Latent	Observed	*B*	SE	|*z*|	*P*	95% CI LB	95% CI UB
PHYS	WEIGHT	.1265	.0562	2.25	.024	.0164	.2367
PHYS	TROUBE	−.5760	.0401	14.36	<.001	−.6546	−.4974
PHYS	ASSWA	−.2623	.0523	5.02	<.001	−.3647	−.1599
PHYS	TROUWO	−.5251	.0422	12.43	<.001	−.6079	−.4423
PHYS	JOINTP	−.4501	.0456	9.87	<.001	−.5395	.−3607
PHYS	AMOVE	.1724	.0544	3.17	.002	.0658	.2790
PHYS	TROUWM	−.6194	.0387	16.01	<.001	−.6953	−.5436
PHYS	LIMITA	−.5392	.0420	12.83	<.001	−.6215	−.4568
PHYS	SEREP	−.4562	.0452	10.09	<.001	−.5449	−.3676
PHYS	PAIN	−.4883	.0436	11.21	<.001	−.5737	−.4029
PHYS	STIFF	−.3261	.0504	6.48	<.001	−.4248	−.2274
PHYS	ASSIST	−.3280	.0500	6.56	<.001	−.4259	−.2300
PHYS	STAYIN	−.3488	.0493	7.07	<.001	−.4455	−.2522
PHYS	INBED	−.3277	.0501	6.54	<.001	−.4260	−.2294
PSY	CESD	.8165	.0212	38.43	<.001	.7748	.8581
PSY	AIMD	.9312	.0152	61.31	<.001	.9014	.9610
PSY	AIMA	.8134	.0214	37.94	<.001	.7714	.8554
PSY	AIMIS	.3454	.0488	7.08	<.001	.2500	.4411
SE	EFFPAIN	.3117	.0588	5.30	<.001	.1965	.4269
SE	EFFSYM	.8502	.0351	24.20	<.001	.7814	.9191
SE	EFFACT	.8805	.0635	13.87	<.001	.7560	1.0049
SE	ARTHINT	−.4691	.0462	10.15	<.001	−.5597	−.3786
SE	ARTHHEL	−.6449	.0406	15.87	<.001	−.7246	−.5652

Note: OV: observed variable; SE: standard error; 95% CI LB: 95% confidence interval lower bound; 95% CI UB: 95% confidence interval upper bound.

**Table 8:** Standardized error covariances within nonmediated structural model.

Latent	First	Second	*r*	SE	|*z*|	*P*	95% CI LB	95% CI UB
PHYS	WEIGHT	AMOVE	−.1017	.0522	1.95	.051	−.2040	.0006
PHYS	WEIGHT	ASSIST	.1363	.0430	3.17	.002	.0520	.2206
PHYS	TROUBE	LIMITA	.2700	.0531	5.07	<.001	.1654	.3737
PHYS	ASSWA	TROUWM	.0998	.0415	2.40	.016	.0184	.1812
PHYS	ASSWA	ASSIST	.4880	.0388	12.58	<.001	.4119	.5640
PHYS	ASSWA	STAYIN	.3551	.0455	7.80	<.001	.2659	.4443
PHYS	ASSWA	INBED	.3365	.0467	7.21	<.001	.2450	.4280
PHYS	TROUWO	TROUWM	.4096	.0467	8.76	<.001	.3180	.5012
PHYS	TROUWO	STAYIN	.1617	.0421	3.84	<.001	.0791	.2442
PHYS	TROUWO	INBED	.1148	.0497	2.31	.021	.0174	.2122
PHYS	JOINTP	SEREP	.3781	.0467	8.10	<.001	.2866	.4695
PHYS	JOINTP	PAIN	.3991	.0459	8.69	<.001	.3091	.4892
PHYS	JOINTP	STIFF	.1974	.0513	3.85	<.001	.0968	.2981
PHYS	AMOVE	ASSIST	−.2214	.0417	5.31	<.001	−.3032	−.1397
PHYS	TROUWM	INBED	.0826	.0507	1.63	.103	−.0167	.1820
PHYS	LIMITA	STIFF	.1048	.0482	2.17	.030	.0103	.1992
PHYS	SEREP	PAIN	.5819	.0359	16.22	<.001	.5116	.6523
PHYS	SEREP	STIFF	.2868	.0489	5.86	<.001	.1909	.3827
PHYS	PAIN	STIFF	.2909	.0490	5.94	<.001	.1949	.3869
PHYS	ASSIST	STAYIN	.4172	.0420	9.94	<.001	.3349	.4995
PHYS	ASSIST	INBED	.2762	.0471	5.86	<.001	.1838	.3685
PHYS	STAYIN	INBED	.3126	.0475	6.58	<.001	.2195	.4057
SE	EFFPAIN	EFFSYM	.2607	.0705	3.70	<.001	.1225	.3989
SE	EFFPAIN	ARTHINT	−.2828	.0486	5.82	<.001	−.3780	−.1876
SE	EFFACT	EFFSYM	.3253	.0649	5.01	<.001	.1981	.4526
	PHYS	SE	−.7172	.0703	10.21	<.001	−.8550	−.5795
	SE	EFFACT	−.4759	.0625	7.61	<.001	−.5985	−.3534
	PSY	SE	−.3549	.0537	6.61	<.001	−.4601	−.2497

Note: OV: observed variable; SE: standard error; 95% CI LB: 95% confidence interval lower bound; 95% CI UB: 95% confidence interval upper bound.

## References

[1] Centers for Disease Control and Prevention Osteoarthritis. http://www.cdc.gov/arthritis/basics/osteoarthritis.htm.

[2] Guccione AA, Felson DT, Anderson JJ (1994). The effects of specific medical conditions on the functional limitations of elders in the Framingham study. *American Journal of Public Health*.

[3] Brault MW, Hootman J, Helmick CG, Theis KA, Armour BS (2009). Prevalence and most common causes of disability among adults—United States, 2005. *Morbidity and Mortality Weekly Report*.

[4] Lawrence RC, Felson DT, Helmick CG (2008). Estimates of the prevalence of arthritis and other rheumatic conditions in the United States. Part II. *Arthritis and Rheumatism*.

[5] Centers for Disease Control and Prevention Osteoarthritis and you. http://www.cdc.gov/features/osteoarthritisplan/.

[6] Centers for Disease Control and Prevention Arthritis meeting the challenge of living well—at a glance. http://www.cdc.gov/chronicdisease/resources/publications/aag/pdf/2013/Arthritis-AAG-2013_508.pdf.

[7] Hochberg MC, Altman RD, April KT (2012). American College of Rheumatology 2012 recommendations for the use of nonpharmacologic and pharmacologic therapies in osteoarthritis of the hand, hip, and knee. *Arthritis Care and Research*.

[8] Kovar PA, Allegrante JP, MacKenzie CR, Peterson MGE, Gutin B, Charlson ME (1992). Supervised fitness walking in patients with osteoarthritis of the knee: a randomized, controlled trial. *Annals of Internal Medicine*.

[9] Evcik D, Sonel B (2002). Effectiveness of a home-based exercise therapy and walking program on osteoarthritis of the knee. *Rheumatology International*.

[10] Sperber NR, Bosworth HB, Coffman CJ (2012). Participant evaluation of a telephone-based osteoarthritis self-management program, 2006–2009. *Preventing Chronic Disease*.

[11] Devos-Comby L, Cronan T, Roesch SC (2006). Do exercise and self-management interventions benefit patients with osteoarthritis of the knee? A metaanalytic review. *Journal of Rheumatology*.

[12] Zhang W, Moskowitz RW, Nuki G (2008). OARSI recommendations for the management of hip and knee osteoarthritis, Part II: OARSI evidence-based, expert consensus guidelines. *Osteoarthritis and Cartilage*.

[13] Christmas C, Andersen RA (2000). Exercise and older patients: guidelines for the clinician. *Journal of the American Geriatrics Society*.

[14] Pope A, Tarlov A (1991). *Disability in America: Toward a National Agenda for Prevention*.

[15] Rejeski WJ, Miller ME, Foy C, Messier S, Rapp S (2001). Self-efficacy and the progression of functional limitations and self-reported disability in older adults with knee pain. *Journals of Gerontology B*.

[16] Lorig K, Chastain RL, Ung E, Shoor S, Holman HR (1989). Development and evaluation of a scale to measure perceived self-efficacy in people with arthritis. *Arthritis and Rheumatism*.

[17] Rejeski WJ, Craven T, Ettinger WH, McFarlane M, Shumaker S (1996). Self-efficacy and pain in disability with osteoarthritis of the knee. *Journals of Gerontology B*.

[18] Bandura A, Ramachaudran VS (1994). Self-efficacy. *Encyclopedia of Human Behavior*.

[19] Bandura A, Friedman H (1998). Self-efficacy. *Encyclopedia of Mental Health*.

[20] Knittle KP, De Gucht V, Hurkmans EJ (2011). Effect of self-efficacy and physical activity goal achievement on arthritis pain and quality of life in patients with rheumatoid arthritis. *Arthritis Care and Research*.

[21] Focht BC, Rejeski WJ, Ambrosius WT, Katula JA, Messier SP (2005). Exercise, self-efficacy, and mobility performance in overweight and obese older adults with knee osteoarthritis. *Arthritis Care and Research*.

[22] Bozoian S, Rejeski WJ, McAuley E (1994). Self-efficacy influences feeling states associated with acute exercise. *Journal of Sport & Exercise Psychology*.

[23] Marks R (2012). Self-efficacy and it application in the treatment of knee osteoarthritis: a critical review. *Rheumatology Reports*.

[24] Harrison AL (2004). The influence of pathology, pain, balance, and self-efficacy on function in women with osteoarthritis of the knee. *Physical Therapy*.

[25] Dekker J, Tola P, Aufdemkampe G, Winckers M (1993). Negative affect, pain and disability in osteoarthritis patients: the mediating role of muscle weakness. *Behaviour Research and Therapy*.

[26] Hill CL, Gill T, Taylor AW, Daly A, Grande ED, Adams RJ (2007). Psychological factors and quality of life in arthritis: a population-based study. *Clinical Rheumatology*.

[27] Scopaz KA, Piva SR, Wisniewski S, Fitzgerald GK (2009). Relationships of fear, anxiety, and depression with physical function in patients with knee osteoarthritis. *Archives of Physical Medicine and Rehabilitation*.

[28] Lampinen P, Heikkinen R, Ruoppila I (2000). Changes in intensity of physical exercise as predictors of depressive symptoms among older adults: an eight-year follow-up. *Preventive Medicine*.

[29] Meenan RF, Gertman PM, Mason JH (1980). Measuring health status in arthritis: the arthritis impact measurement scales. *Arthritis and Rheumatism*.

[30] Kaplan RM, Anderson JP, Spilker B (1990). The general health policy model: an integrated approach. *Quality of Life Assessments in Clinical Trials*.

[31] Radloff LS (1977). The CES-D scale: a self-report depression scale for research in the general population. *Applied Psychological Measurement*.

[32] Stein MJ, Wallston KA, Nicassio PM (1988). Factor structure of the Arthritis Helplessness Index. *Journal of Rheumatology*.

[33] Bentler PM (2007). On tests and indices for evaluating structural models. *Personality and Individual Differences*.

[34] Arnstein P, Caudill M, Mandle CL, Norris A, Beasley R (1999). Self efficacy as a mediator of the relationship between pain intensity, disability and depression in chronic pain patients. *Pain*.

